# Analysis of the subject-object effects and chain mediation of psychosocial factors in couples during late pregnancy on depression: a cross-sectional study of pregnant women and their spouses

**DOI:** 10.3389/fpubh.2026.1948517

**Published:** 2026-09-28

**Authors:** Yunhui Shi, Han Wang, Wenhui Han, Aixia Zhang

**Affiliations:** 1 School of Nursing, Nanjing Medical University, Jiangsu, China; 2 Nursing Department, Women's Hospital of Nanjing Medical University (Nanjing Women and Children's Healthcare Hospital), Jiangsu, China; 3 Nanjing University of Chinese Medicine, Nanjing, China

**Keywords:** couples, depression, late pregnancy, psychosocial factors, the subject-object relationship

## Abstract

**Objective:**

To examine, from a dual-perspective approach, the actor and partner associations of couples' perceptions of stress, rumination, self-efficacy, self-disclosure, and family intimacy with depressive symptoms during late pregnancy, and to test the chain mediation mechanism of “stress—family intimacy—rumination/self-disclosure—depression.

**Methods:**

From July 2025 to May 2026, 1,331 pairs of pregnant women and their spouses attending prenatal checkups at the outpatient clinic of a Grade III Level A hospital in Nanjing were selected as study participants using convenience sampling. The Patient Health Questionnaire (PHQ-9), the Perceived Stress Scale (PSS), the Rumination Scale (RRS), the General Self-Efficacy Scale, the Distress Disclosure Scale, and the Family Affection and Cohesion Scale (FACES II) were used to collect data. Data analysis was performed using paired-sample *t*-tests, multiple linear regression, and the Agent-Patient Interdependence Model (APIM), with the Bootstrap method used to test for mediating effects.

**Results:**

(1) Positive Detection Rate for Symptoms of Depression among women was 18.3%, and among their spouses, it was 16.7%. (2) Spouses' perceived stress and ruminative thinking were significantly higher than those of pregnant women (both *P* < 0.001), while pregnant women's self-disclosure, self-efficacy, family closeness, and adaptability were all significantly higher than those of their spouses (all *P* < 0.001). (3) Multiple linear regression analysis revealed that ruminative thinking (pregnant women: β = 0.337; spouses: β = 0.282) and perceived stress (pregnant women: β = 0.153; spouses: β = 0.240) were common core predictors of depression in both partners. (4) Univariate APIM analysis revealed that all subject effects were significant (*P* < 0.001), while only the spouses' rumination (β = 0.067, *P* = 0.003) and perceived stress (β = 0.052, *P* = 0.028) exhibited significant object effects. (5) The chained mediation model showed that the full pathway “stress → reduced family intimacy → increased rumination → worsened depression” exhibited significant indirect associations for both spouses, with the proportion of indirect effect higher in the pregnant women group (49.70%) than in the spouse group (35.66%); the pathway “stress → reduced family intimacy → decreased self-disclosure → increased depression” was also significant but showed a weaker effect. Cross-mediation analysis indicated that stress in one partner was indirectly associated with the other partner's depression levels, with this association occurring through the perceived family intimacy of the partner.

**Conclusion:**

Depression among couples in late pregnancy is primarily driven by intra-individual associations, with rumination and perceived stress serving as core correlates; family intimacy may act as a key correlate linking stress to depression and cross-individual spillover patterns. Family intimacy was indirectly associated with depression levels in both partners, and these associations operated through rumination and self-disclosure levels. Clinical interventions should focus primarily on individual psychological adjustment to reduce stress and ruminative thinking in both the expectant mother and the father, while also emphasizing enhanced communication to improve marital intimacy and alleviate depressive symptoms in both partners.

## 1 Introduction

Perinatal depression (PND) is one of the most common mental health issues among pregnant women and new mothers worldwide. It not only affects the quality of life of pregnant women themselves but is also closely associated with adverse outcomes such as preterm birth, low birth weight, maternal-infant attachment disorders, and abnormalities in children's neuropsychological development (1–3). In recent years, a large body of research has confirmed that prenatal depression is not only the most important predictor of postpartum depression (4) but also a significant risk factor affecting the mental health of the entire family. Consequently, early identification and intervention for perinatal mental health have become critical issues in the field of public health (5).

The third trimester is a peak period for perinatal psychological issues. On the one hand, physiological changes are most pronounced during late pregnancy—physical discomfort caused by rapid fetal growth, sleep disturbances, and anxiety stemming from the uncertainty of impending childbirth are all sources of psychological stress (6, 7). On the other hand, during this stage, both partners must simultaneously manage multiple tasks, such as deciding on a delivery plan, preparing for parenting, and adjusting financial and housing arrangements. This “multiple-role overlap” creates a heavy stress burden that poses challenges for both partners. More importantly, prenatal depression has been identified as the strongest predictor of postpartum depression (4), and during the third trimester and the postpartum period, women are more susceptible to stress, anxiety, and depression (8). Stress and depression scores during late pregnancy are associated with postpartum depression and serve as strong predictors of postpartum depression (9, 10). For example, A Chinese study of 1,196 participants further reported a correlation coefficient as high as 0.706 between late-pregnancy depression and postpartum depression (6). Therefore, focusing on the psychosocial factors of both partners during late pregnancy not only helps elucidate the mechanisms underlying depression in both partners during this stage and develop clinical intervention strategies for late-pregnancy depression in couples, but also contributes to reducing the incidence of postpartum depression.

Compared to expectant mothers, the mental health of expectant fathers has long been overlooked. However, as family roles shift and financial and caregiving responsibilities increase, fathers also experience significant psychological stress during pregnancy. A meta-analysis shows that the prevalence of prenatal depression among fathers is approximately 10%, and it is significantly comorbid with depression in expectant mothers (11–13). Existing research has found that stress, depression, and marital relationships among couples exhibit significant interdependence; when one partner experiences depression, it can affect the other's mental health through mechanisms such as emotional contagion, stress spillover, and family interactions (14–16). Therefore, studying perinatal depression solely from an individual perspective may underestimate the complex interactions between partners.

The Actor–Partner Interdependence Model (APIM) can simultaneously estimate actor effects and partner effects and has gradually become an important method for analyzing couple-level bivariate data in recent years (17). Existing APIM studies have found that prenatal stress, marital satisfaction, and social support all exhibit certain actor effects, while findings regarding partner effects remain inconsistent. Liu et al. (18) suggest that prenatal stress primarily influences one's own depression through actor effects, whereas another study found that stress can indirectly affect a partner's depression via marital satisfaction (19). Tang et al. (20) further pointed out that shared depression in couples is influenced not only by individual risk factors but also by the combined effect of the quality of the marital relationship. These studies suggest that different psychosocial factors may influence depression in both partners through distinct pathways, and the underlying interactive mechanisms require further clarification.

According to Stress and Coping Theory, when individuals face stressful events, family resources and cognitive coping strategies jointly determine psychological outcomes. Stress is associated with reduced protective resources such as family closeness, and is concurrently associated with higher levels of negative cognitions such as rumination and lower levels of positive coping behaviors such as self-disclosure; these factors are in turn associated with higher depression levels. However, current perinatal research largely focuses on single variables such as stress, marital satisfaction, or social support, lacking a systematic integration of various psychosocial factors—including perceived stress, family functioning, ruminative thinking, self-disclosure, and self-efficacy. In particular, there is a lack of research exploring subject-object interactions and chained mediation mechanisms based on the APIM framework.

The aforementioned studies suggest that the interaction patterns of psychosocial factors between spouses may vary across samples. In summary, few existing studies have compared the differences in pathways within the same model between pregnant women and expectant fathers; intervention strategies often “prioritize mothers over fathers,” and there is a lack of targeted understanding of the unique stress–cognition–depression pathway specific to fathers. Previous perinatal APIM studies have largely focused on single variables such as stress, marital satisfaction, or social support, lacking an integrated examination of the complete psychosocial transmission chain “perceived stress → family intimacy → coping strategies → depression” within a single framework. At the mechanistic level, although a small number of studies suggest that rumination or self-disclosure may be associated with perinatal depression, there is still no empirical answer based on dyadic data regarding “what role family intimacy plays between stress and depression—whether it is a simple direct protective factor or acts indirectly by shaping the cognitive processing patterns (rumination/disclosure) of both partners.” Based on stress coping theory (“stressor—resource—coping strategy—outcome”), this study hypothesizes that perceived stress is associated with depression through pathways involving family intimacy, which in turn are associated with negative (rumination) or positive coping strategies (self-disclosure). This study aims to systematically analyze the subject-object effects of the aforementioned psychosocial factors on spousal depression using a large sample of 1,331 couples in late pregnancy, and to test the chained mediation mechanism of “stress—family functioning—coping strategies—depression,” thereby providing a theoretical basis for dual-track interventions for perinatal depression.

This study constructs two parallel chain-mediation APIM models, using rumination (negative cognition) and self-disclosure (positive behavior) as mediating variables, respectively, to systematically compare: (1) the relative effect sizes of the two coping strategies in the stress-to-depression transmission pathway for both spouses; (2) whether the “hub” role of family intimacy in the stress → coping style → depression chain exhibits cross-subject spillover effects; (3) gender-specific patterns among expectant mothers and fathers within the complete chain of pathways. This research design—featuring a “single theoretical framework, parallel comparison of dual pathways, and couple-level interaction”—is the first of its kind in the field of perinatal mental health and can provide theoretical targets for subsequent precise, gender-differentiated joint interventions for couples.

Specifically, by distinguishing between subject effects and object effects, this study addresses a long-standing clinical question: Does stress in one partner influence the other's depression through “direct emotional contagion,” or is it transmitted indirectly by “deteriorating the quality of the family relationship as jointly perceived by both partners”? This distinction provides direct guidance for selecting intervention targets.

## 2 Methods

### 2.1 Study design and setting

This study was a cross-sectional study conducted in the outpatient department of a tertiary hospital in Nanjing, China, from July 2025 to May 2026.

### 2.2 Participants

Using convenience sampling, pregnant women undergoing outpatient prenatal checkups at a Grade III Class A hospital in Nanjing and their spouses were selected as study participants. Inclusion criteria: (1) gestational age ≥28 weeks; (2) both spouses provided informed consent and voluntarily participated; (3) ability to complete the questionnaire independently. Exclusion criteria: (1) history of severe pregnancy complications or mental illness in the pregnant woman; (2) inability of either spouse to cooperate with the survey.

A total of 1,652 questionnaires were distributed. Questionnaires with excessively short response times or missing data were excluded (if either spouse did not meet the criteria, the paired data were excluded); ultimately, 1,331 valid paired questionnaires were included in the analysis. The study obtained written informed consent from all participants. Following eligibility screening and informed consent, trained researchers detailed the study objectives and procedures. Participants completed self-administered questionnaires on-site under direct supervision. Spouses completed questionnaires independently in separate rooms without communication, with immediate collection upon completion to ensure data integrity. We emphasized anonymity and confidentiality throughout, enabling participants to express feelings freely and enhancing data reliability.

### 2.3 Outcome variables and methods of assessment

#### 2.3.1 Socio-demographic information

A general information questionnaire was designed in-house, covering age, educational level, average monthly household income per capita, number of pregnancies and births, family planning status, satisfaction with sleep quality, and marital satisfaction.

#### 2.3.2 The Patients' Health Questionnaire (PHQ-9)

PHQ-9 consists of nine items. The PHQ-9 was developed based on the depressive symptoms outlined in the American Diagnostic and Statistical Manual of Mental Disorders, Fourth Edition (DSM-IV) to assess the severity of depressive symptoms over the past 2 weeks. It uses a four-point rating scale ranging from 0 to 3, with a total score ranging from 0 to 27; a higher score indicates more severe depressive symptoms (21). In accordance with the Guidelines for the Prevention and Treatment of Perinatal Depression issued by the National Health Commission of China and recommendations from previous large-scale perinatal studies, this study used a PHQ-9 total score of ≥5 as the screening cutoff for the presence of depressive symptoms (i.e., a positive result for depressive symptoms) (22). In this study, the Cronbach's alpha for the Pregnant Women's Version was 0.796, and for the Spouses' Version, it was 0.831.

#### 2.3.3 Perceived Stress Scale (PSS)

This scale consists of 14 items. The total perceived stress score is calculated by summing all items; a higher score indicates a higher level of stress subjectively experienced by the individual in daily life. It is used to assess an individual's perception of stress (23) and employs a five-point rating scale ranging from 0 to 4. The alpha coefficient for the Pregnant Women's Version is 0.863, and for the Spouses' Version, it is 0.813.

#### 2.3.4 The Ruminative Response Scale (RRS)

RRS consists of 22 items. It was developed by Nolen-Hoeksema et al. (24) in 2008 and adapted into Chinese by Han and Yang (25) to assess individuals' responses to life events. The scale comprises three dimensions—symptomatic rumination, obsessive thinking, and reflective rumination—with a total of 22 items. It uses a 4-point Likert scale, with scores ranging from 1 to 4 for responses ranging from “Never” to “Always,” resulting in a total score ranging from 22 to 88. A higher score indicates more severe ruminative thinking. The alpha coefficient for the Pregnant Women Version is 0.963, and for the Spouse Version, it is 0.958.

#### 2.3.5 The General Self-Efficacy Scale (GSES)

GSES consists of 10 items and assesses an individual's level of confidence in coping with difficulties and setbacks. It uses a four-point rating scale ranging from 1 to 4; a higher total score indicates a higher level of self-efficacy (26). The alpha coefficient for the Pregnant Women's Version is 0.946, and for the Spouses' Version, it is 0.929.

#### 2.3.6 The Distress Disclosure Index (DDI)

The DDI was translated into Chinese by Li Xinmin, and this Chinese version was used in the study by Yu Ya et al. has been used in China to measure the degree of distress disclosure among patients (27) and consists of 12 items. The scale uses a 1–5-point rating scale, with a total score ranging from 12 to 60. A higher score indicates a stronger willingness to disclose distress; scores of 12–29 represent a low level, 30–44 a moderate level, and 45–60 a high level. The Cronbach's alpha for the pregnant women version is 0.931, and for the spouse version, it is 0.923.

#### 2.3.7 The Family Affection and Coping Scale (FACES II)

We used the Chinese version of the second edition of the Family Adaptability and Cohesion Evaluation Scales (FACES II), translated and revised by Fei et al. (28) and originally developed by Olson et al. (38). The scale consists of 30 items across two subscales, assessing the emotional bonds among family members and the family system's ability to cope with change, using a five-point rating scale from 1 to 5. The Family Affection subscale contains 16 items, and the Family Coping subscale contains 14 items. Items on the scale are scored on a 1–5 scale, representing “never,” “rarely,” “sometimes,” “often,” and “always,” respectively. The Cronbach's alpha for the Family Intimacy dimension was 0.888 for the pregnant women's version and 0.864 for the spouses' version; for the Family Adaptation dimension, it was 0.898 for the pregnant women's version and 0.884 for the spouses' version.

#### 2.3.8 Validity and reliability testing of scales

Reliability and validity analyses were conducted on the scales. First, regarding reliability, Cronbach's alpha is a commonly used measure of internal consistency. Generally, α ≥ 0.9 indicates very good reliability, 0.8 ≤ α < 0.9 indicates good reliability, and 0.7 ≤ α < 0.8 indicates acceptable reliability. The reliability coefficients for all scales used in this study were greater than 0.7; therefore, the overall stability and consistency of the scales met the standards. Regarding validity, the KMO values for all scales were greater than 0.8, indicating good validity.

### 2.4 Ethical considerations

The study adhered to the ethical principles of the Declaration of Helsinki and received ethical approval from the institutional review board at the Nanjing Medical University Affiliated Maternal and Child Health Hospital (2022KY-137). Before data collection, the researcher detailed the study's purpose, methods, and significance to the eligible participants. Participants were also given the opportunity to ask any questions about the study before fully agreeing to participate. After asking for consent, all participants signed informed consent forms. All participants had the right to withdraw from the study at any time.

## 3 Statistical analyses

Data analysis was performed using SPSS26.0 (IBM Corp., Armonk, NY, USA) and AMOS26.0 (IBM Corp., Armonk, NY, USA). (1) Descriptive statistics: frequency, percentage, mean ± standard deviation. (2) Reliability and validity analysis: Cronbach's α coefficient and KMO test. (3) Tests of differences: Paired-sample *t*-tests were used to compare differences in scale scores between spouses. (4) Analysis of influencing factors: One-way ANOVA was used to screen potential influencing factors, and multiple linear regression analysis was used to identify independent risk factors included in the model. (5) Subject-Object Interdependence Model (APIM): a univariate APIM, a multivariate integrated APIM, and a chained mediation APIM were constructed. Model fit indices included χ^2^/df, GFI, CFI, and RMSEA. The mediation effects were tested using the Bootstrap method (5,000 repeated samples), with a 95% confidence interval not containing 0 indicating a statistically significant difference. The significance level was set at α = 0.05.

## 4 Results

### 4.1 Demographic features of the participants

Descriptive statistics were performed on general characteristics of pregnant women and their spouses (age, educational level, parity, planned mode of delivery, sleep patterns, etc.); specific details are presented in Table 1. Among the pregnant women, the majority were aged 30–34 (49.7%), most had a college or bachelor's degree (73.2%), 78.4% were primiparous, 80.1% had planned pregnancies, and 43.7% reported satisfactory sleep quality.

**Table 1:** General characteristics of pregnant women (*N* = 1,331).

Variable	Category	Frequency	Percentage
Age	18–24 years	26	2
	25–29 years	365	27.4
	30–34 years	661	49.7
	≥35 years	279	21
Education level	Junior high school or below	31	2.3
	High school or technical secondary school	48	3.6
	College or bachelor's degree	974	73.2
	Graduate degree or above	278	20.9
Monthly household income per capita	≤ 5,000 CNY	152	11.4
	5,001–10,000 CNY	628	47.2
	≥10,001 CNY	551	41.4
Residence	Owned house in city	1,080	81.1
	Rented house in city	200	15
	Rural area	51	3.8
Satisfaction with sleep quality	Satisfied	582	43.7
	Fair	686	51.5
	Dissatisfied	63	4.7
Concern about personal appearance changes	Concerned	443	33.3
	Moderate	731	54.9
	Not concerned	157	11.8
Only child	Yes	604	45.4
	No	727	54.6
Marital quality	Very good	986	74.1
	Good	309	23.2
	Fair	35	2.6
	Poor	1	0.1
Planned pregnancy	Planned	1,066	80.1
	Unplanned	265	19.9
	Natural conception	1,184	89
	Assisted conception	147	11
Parity	Primipara	1,043	78.4
	Multipara	288	21.6
History of adverse pregnancy outcomes	Yes	357	26.8
	No	974	73.2
Planned mode of delivery	Vaginal delivery	978	73.5
	Cesarean section	353	26.5
Readiness for newborn care	Adequate	939	70.5
	Fair	381	28.6
	Inadequate	11	0.8
Chronic disease	Yes	67	5
	No	1,264	95
Pregnancy complications	Yes	353	26.5
	No	978	73.5

The spouses' ages were predominantly in the 30–34 age group (50.4%), most had a college or bachelor's degree (67.0%), and 94.4% were employed. Specific details are shown in Table 2.

**Table 2:** General characteristics of fathers (*N* = 1,331).

Variable	Category	Frequency	Percentage
Age	18–24 years	14	1.1
	25–29 years	278	20.9
	30–34 years	671	50.4
	≥35 years	368	27.6
Education level	Junior high school or below	30	2.3
	High school or technical secondary school	68	5.1
	College or bachelor's degree	892	67
	Graduate degree or above	341	25.6
Occupation	Employed	1,256	94.4
	Part-time	17	1.3
	Unemployed/laid-off	58	4.4
Monthly income	≤ 5,000 CNY	133	10
	5,001–10,000 CNY	482	36.2
	≥10,000 CNY	716	53.8
Only child	Yes	743	55.8
	No	588	44.2
Planned pregnancy	Planned	1,118	84
	Unplanned	213	16
Father's expectation of fetal sex	Expect	493	37
	Neutral	564	42.4
	Not expect	274	20.6
Satisfaction with sleep quality	Satisfied	801	60.2
	Fair	499	37.5
	Dissatisfied	31	2.3
Marital quality	Very good	1,144	86
	Good	169	12.7
	Fair	18	1.4
Readiness for newborn care	Adequate	1,094	82.2
	Fair	231	17.4
	Inadequate	6	0.5
Alcohol consumption	Never	432	32.5
	Occasionally	844	63.4
	Often	55	4.1
Smoking	Never	903	67.8
	Occasionally	251	18.9
	Often	177	13.3

### 4.2 Positive detection rate for depressive symptoms among couples and Scale Scores

Among pregnant women, 244 had a PHQ-9 score of 5 or higher, with a depression prevalence of 18.3%; among spouses, 222 had a PHQ-9 score of 5 or higher, with a depression prevalence of 16.7%. Specific scores for each scale are shown in Table 3.

**Table 3:** Descriptive statistics.

Variable	*N*	Minimum	Maximum	Mean	Std. deviation
Pregnant women's PHQ-9 scores	1,331	0	25	2.44	2.729
Fathers' PHQ-9 scores	1,331	0	25	2.21	2.69
Pregnant women's rumination	1,331	22	88	29.89	8.904
Fathers' rumination	1,331	22	88	34.07	10.502
Pregnant women's self-efficacy	1,331	11	40	30.05	5.861
Fathers' self-efficacy	1,331	10	40	29.23	5.575
Pregnant women's perceived stress	1,331	14	57	28.7	7.749
Fathers' perceived stress	1,331	14	53	30.01	7.065
Pregnant women's self-disclosure	1,331	17	60	48.11	8.544
Fathers' self-disclosure	1,331	12	60	39.15	9.466
Pregnant women's family cohesion	1,331	17	80	63.31	9.465
Fathers' family cohesion	1,331	29	80	61.63	9.187
Pregnant women's family adaptability	1,331	20	70	55.17	7.962
Fathers' family adaptability	1,331	19	70	53.88	8.024
Valid *N* (listwise)	1,331				

### 4.3 Differences in scores for various variables between spouses

A paired-sample *t*-test revealed (Supplementary Table S1) that pregnant women's depression scores were slightly higher than those of their spouses (*P* = 0.023); spouses' perceived stress and rumination were significantly higher than those of pregnant women (both *P* < 0.001); whereas pregnant women's self-disclosure, self-efficacy, family intimacy, and adaptability were all significantly higher than those of their spouses (all *P* < 0.001). These findings suggest that women are more proactive in emotional expression and perceptions of family functioning, while men are more pronounced in stress perception and negative cognitions.

### 4.4 Multiple linear regression analysis of factors influencing depression

With the pregnant women's PHQ-9 scores as the dependent variable, variables that were statistically significant in the univariate analysis were included in a multiple linear regression model, for details on the univariate analysis, see Appendix A1. To examine the independent associations of psychosocial factors with depressive symptoms while controlling for potential confounders, we performed hierarchical multiple linear regression analyses. The hierarchical regression analyses aimed to identify the independent psychosocial correlates of depressive symptoms after adjusting for demographic and clinical covariates. Supplementary Table S2 presents the results for maternal depression, and Supplementary Table S3 presents the results for paternal depression. For both tables, Model 1 included only the significant covariates from univariate analyses (i.e., control variables); subsequent models added the key psychosocial predictors of interest to test their unique contributions and the change in explained variance (Δ*R*^2^). Evaluating their incremental predictive validity over and above the covariates. Table S2 presents the hierarchical regression results for maternal depression. After controlling for demographic covariates (Model 1, *R*^2^ = 0.236), the addition of the psychosocial block in Model 2 significantly increased the explained variance to 47.6% (Δ*R*^2^ = 0.240, *P* < 0.001). The results showed that ruminative thinking among pregnant women was the strongest predictor (β = 0.337), followed by sleep dissatisfaction and perceived stress; higher educational attainment, family closeness, and self-efficacy served as protective factors. It is worth noting that the father's ruminative thinking also influenced the pregnant women's depression; see Supplementary Table S2 for the specific model.

Fathers' perceived stress and ruminative thinking are the primary predictors of paternal depression, while family closeness and adaptiveness serve as protective factors; see Supplementary Table S3 for the specific models. Model 3 builds upon Model 2 by adding ruminative thinking as a primary psychological factor, causing the effect of paternal self-disclosure on depression to change from significant (*t* = −2.115, *P* = 0.031) to non-significant (*P* = 0.117). Supplementary Table S3 presents the hierarchical regression results for paternal depression. After adjusting for covariates (Model 1, *R*^2^ = 0.205), the addition of stress, self-disclosure, and family functioning in Model 2 raised *R*^2^ to 0.370 (Δ*R*^2^ = 0.165), and the further inclusion of rumination in Model 3 raised it to 0.412 (Δ*R*^2^ = 0.042). Notably, the significant effect of paternal self-disclosure observed in Model 2 (β = −0.051, *P* = 0.031) became non-significant after rumination entered Model 3 (β = −0.036, *P* = 0.117), suggesting that the protective effect of self-disclosure may be partially accounted for by its association with reduced rumination.

### 4.5 The subject-object interdependence model

#### 4.5.1 Single-variable APIM

A single-variable APIM was constructed using perceived stress, self-disclosure, self-efficacy, family closeness, family adaptability, rumination, and the composite scores for family closeness and adaptability as independent variables, and depression scores for both spouses as the dependent variable. Detailed results of the models are presented in Supplementary Table S4.

Main effects: main effects for all variables were significant (*P* < 0.001), indicating that each individual's own psychosocial factors significantly influenced their own depression levels. Among these, ruminative thinking (pregnant woman: β = 0.555; spouse: β = 0.472) and perceived stress (pregnant woman: β = 0.503; spouse: β = 0.515) had the strongest effects.

Object effects: the vast majority of object effects were not significant (*P* > 0.05). Only the spouse's ruminative thinking (β = 0.067, *P* = 0.003) and the spouse's perceived stress (β = 0.052, *P* = 0.028) significantly and positively predicted the pregnant woman's depression; that is, the greater the spouse's stress and the more severe the spouse's ruminative thinking, the higher the pregnant woman's depression levels.

#### 4.5.2 Multivariate integrated APIM

Therefore, based on the “stressor–resource–coping–outcome” framework of stress coping theory, this study constructed two subject-object interdependent mediation models: “stress–family closeness and adaptiveness–rumination (negative coping strategy)–depression” and “stress–family closeness and adaptiveness–self-disclosure (positive coping strategy)–depression.”

##### 4.5.2.1 Chain mediation model 1 (negative coping): stress—family intimacy—rumination—depression

Based on stress coping theory, a chain mediation model was constructed as follows: “Perceived Stress → Family Intimacy and Adaptiveness → Rumination → Depression,” as shown in Figure 1. The model exhibited excellent fit (CMIN/DF = 0.568, RMSEA = 0.000, CFI = 1.000).

**Figure 1:**
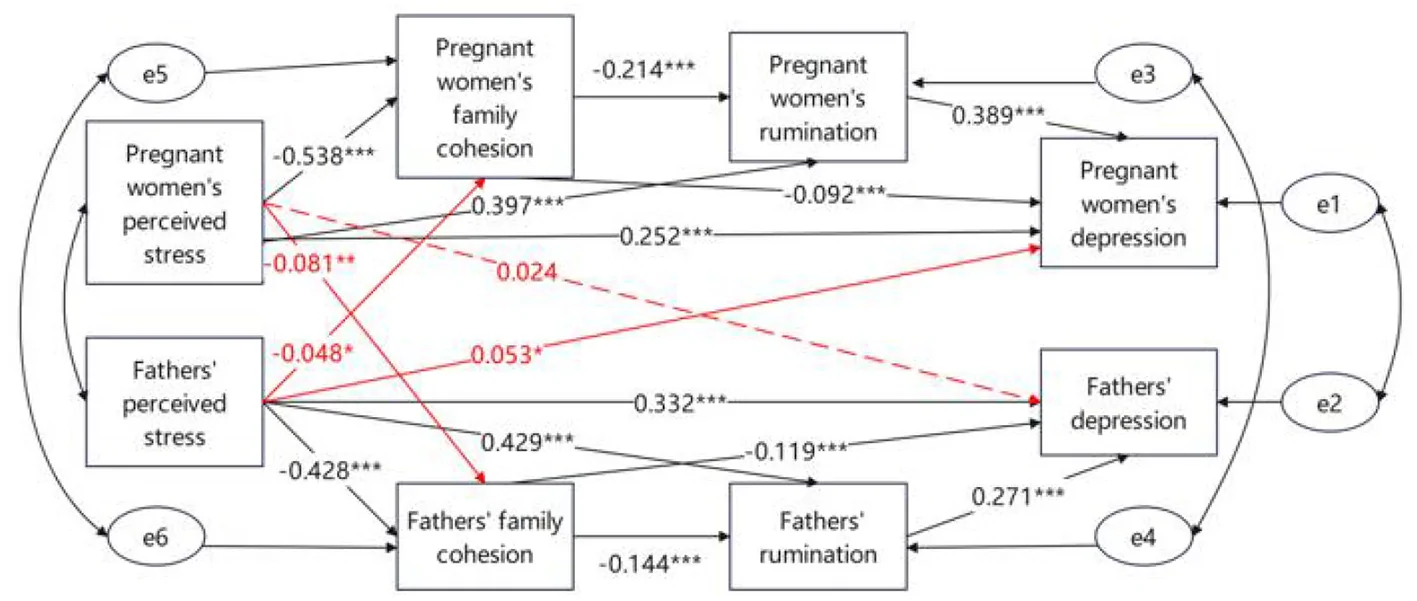
Model 1 of mutual interdependence between subject and object. ^*^p < 0.05, ^**^p < 0.01, ^***^p < 0.001.

Decomposition of the total effect in Model 1 (Table 4): The total effect of stress on depression was significant for both spouses (spouse β = 0.516, pregnant woman β = 0.501), with the proportion of the effect mediated by the pregnant woman group (49.70%) being higher than that in the spouse group (35.66%). Cross-mediation: the total effect of the pregnant woman's stress on the spouse's depression was significant (β = 0.036), fully mediated through the impairment of the spouse's family intimacy (indirect β = 0.013, 95% CI does not include 0); The total effect of Spousal Stress → Pregnant Woman's Depression was significant (β = 0.062); both the direct effect (β = 0.053, *P* = 0.025) and the indirect effect (β = 0.008) were significant, indicating partial mediation.

**Table 4:** Decomposition of total effects in the serial mediation model of stress → family cohesion → rumination → depression.

Group	Effect path	Total effect β	Direct effect β	Direct effect *P*	Indirect effect β	Indirect effect *P*	Mediation proportion (%)	Mediation type
Father group	Fathers' perceived stress → Fathers' depression	0.516	0.332	< 0.001	0.184	< 0.001	35.70%	Partial mediation
Pregnant women group	Pregnant women's perceived stress → Pregnant women's depression	0.501	0.252	< 0.001	0.249	< 0.001	49.70%	Partial mediation
Cross-group	Pregnant women's perceived stress → Fathers' depression	0.036	0.024	0.319	0.013	< 0.001	36.10%	Complete mediation
Cross-group	Fathers' perceived stress → Pregnant women's depression	0.062	0.053	0.025	0.008	0.023	12.90%	Partial mediation

All β values are standardized coefficients. Complete mediation indicates that the indirect effect is significant while the direct effect is not significant (P > 0.05). Partial mediation indicates that both the direct and indirect effects are significant.

Model path coefficients (Supplementary Table S5): stress levels in both spouses significantly and negatively predicted their own perceived family closeness (spouse β = −0.428, pregnant woman β = −0.538); Family closeness negatively predicted ruminative thinking (spouse β = −0.144, pregnant woman β = −0.214); ruminative thinking positively predicted depression (spouse β = 0.271, pregnant woman β = 0.389). Cross-subject effects: maternal stress → Spousal perceived family closeness (β = −0.081, *P* = 0.001); Spousal stress → Maternal perceived family closeness (β = −0.048, *P* = 0.038), suggesting that stress “spills over” from one partner to the other, impairing the other's perception of the family relationship; the direct effect of spousal stress on maternal depression was significant (β = 0.053, *P* = 0.014).

Summary: the 95% Bootstrap confidence intervals for all 10 specific pathways did not include 0, and *P* < 0.05 (all significant). Comparing the effect sizes of the 10 different mediating pathways (Table 5), the “stress → rumination → depression” pathway contributed the most (63.8% in the fathers' group and 62.1% in the pregnant women's group), the complete chain mediation path “Stress → Intimacy → Rumination → Depression” was significant for both spouses (β = 0.017 in the fathers' group; β = 0.045 in the pregnant women's group). Specifically:

1. In both the fathers' and pregnant women's groups, the “Stress → Rumination → Depression” path accounted for more than 60% of the total indirect effect, making it the primary mediating pathway through which stress influences one's own depression.
2. The contribution of the simple intimacy mediation pathways (P2, P5) ranged from 20 to 27%, representing secondary pathways.
3. The contribution of the chained mediation pathways (P3, P6) was the smallest (approximately 9%−18%), indicating that the complex transmission effect of intimacy influencing depression via rumination is relatively weak.
4. In the cross-group analysis, the contribution rate of the simple intimacy mediation (P7, P9) (25%−37.5%) was higher than that of the chained mediation (P8, P10, 12.5%−25%), suggesting that direct damage to intimate relationships is the primary mechanism of the stress spillover effect.

**Table 5:** Standardized indirect effects and contribution rates of specific indirect paths (including total indirect effect).

Group	Code	Specific path	Standardized indirect effect	Path contribution rate (%)
Father group	P1	Perceived stress → Rumination → Depression	0.116	63.8
	P2	Perceived stress → Cohesion → Depression	0.051	27.5
	P3	Perceived stress → Cohesion → Rumination → Depression (serial)	0.017	8.7
Total indirect effect (sum)			0.184	100
Pregnant women group	P4	Perceived stress → Rumination → Depression	0.154	62.1
	P5	Perceived stress → Cohesion → Depression	0.049	19.5
	P6	Perceived stress → Cohesion → Rumination → Depression (serial)	0.045	18.4
Total indirect effect (sum)			0.248	100
Cross-group	P7	Pregnant women's perceived stress → Fathers' cohesion → Fathers' depression	0.01	37.5
	P8	Pregnant women's perceived stress → Fathers' cohesion → Fathers' rumination → Fathers' depression (serial)	0.003	12.5
	P9	Fathers' perceived stress → Pregnant women's cohesion → Pregnant women's depression	0.004	25
	P10	Fathers' perceived stress → Pregnant women's cohesion → Pregnant women's rumination → Pregnant women's depression (serial)	0.004	25
Total indirect effect (sum)			0.021	100

All indirect effects are standardized. Path contribution rate = (specific indirect effect / total indirect effect) × 100%. “Cohesion” refers to family cohesion; “rumination” refers to ruminative thinking.

##### 4.5.2.2 Chain mediation model 2 (positive coping): stress—family intimacy—self-disclosure—depression

Based on stress coping theory, a chain mediation model was constructed as follows: “Perceived Stress → Family Intimacy and Adaptation → Self-Disclosure → Depression,” as shown in Figure 2. The model exhibited excellent fit (CMIN/DF = 0.845, RMSEA = 0.000, CFI = 1.000).

**Figure 2:**
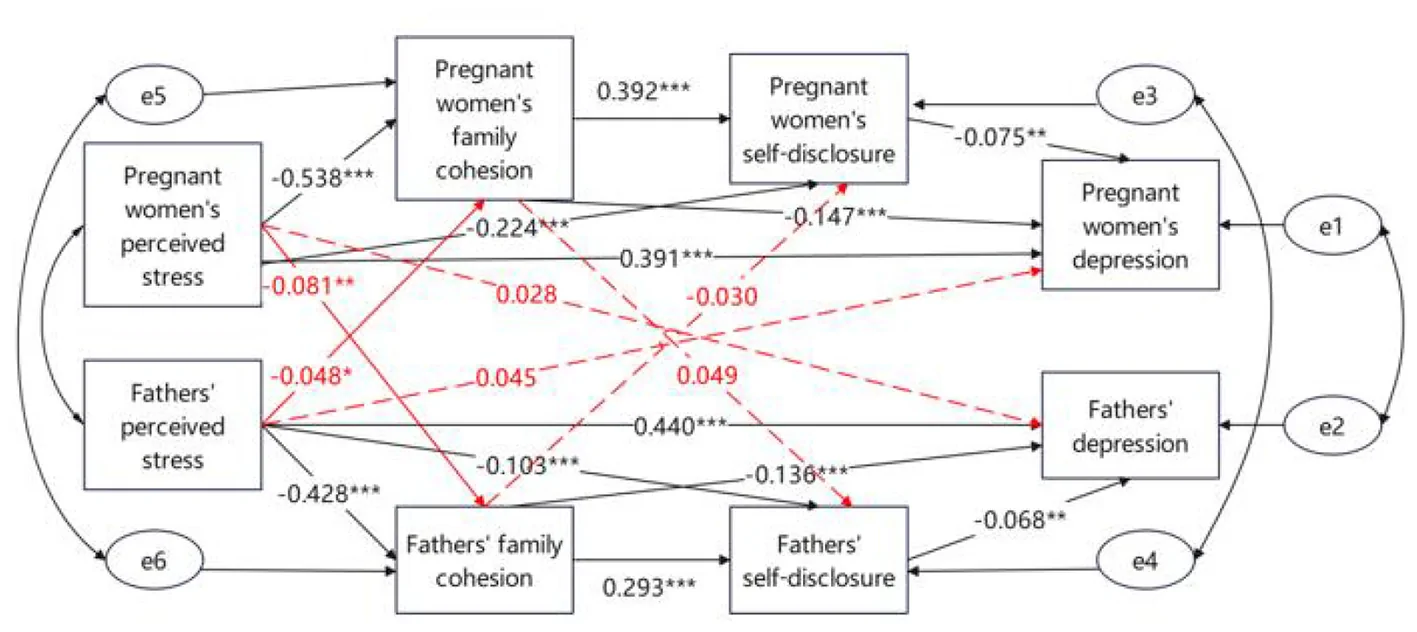
Model 2 of the subject-object interdependence mediation model. ^*^p < 0.05, ^**^p < 0.01, ^***^p < 0.001.

Decomposition of the total effects in Model 2 (Table 6): The total effects of stress on depression were significant (spouse β = 0.514, pregnant women β = 0.503). The proportion of mediation was higher in the pregnant women group (22.27%) than in the spouse group (14.40%), but the overall magnitude of the mediating effect was significantly lower than in Model 1. All cross-path effects were fully mediated.

**Table 6:** Decomposition of total effects in the serial mediation model of stress → family cohesion → self-disclosure → depression.

Group	Effect path	Total effect β	Direct effect β	Direct effect *P*	Indirect effect β	Indirect effect *P*	Mediation proportion (%)	Mediation type
Father group	Fathers' perceived stress → Fathers' depression	0.514	0.44	< 0.001	0.074	< 0.001	14.40%	Partial mediation
Pregnant women group	Pregnant women's perceived stress → Pregnant women's depression	0.503	0.391	< 0.001	0.112	< 0.001	22.30%	Partial mediation
Cross-group	Pregnant women's perceived stress → Fathers' depression	0.043	0.028	0.226	0.015	< 0.001	34.90%	Complete mediation
Cross-group	Fathers' perceived stress → Pregnant women's depression	0.053	0.045	0.055	0.006	0.018	11.30%	Complete mediation

Complete mediation indicates that the indirect effect is significant while the direct effect is not significant (P > 0.05); partial mediation indicates that both direct and indirect effects are significant.

Path coefficients (Supplementary Table S6): Family closeness positively predicts self-disclosure (spouse β = 0.293, pregnant woman β = 0.392), and self-disclosure negatively predicts depression (spouse β = −0.068, pregnant woman β = −0.075). The negative predictive effect of stress on self-disclosure (spouse β = −0.103, pregnant woman β = −0.224) was much weaker than the positive effect of stress on rumination in Model 1, and the predictive effect of self-disclosure on depression was also much weaker than that of rumination.

Summary: The Bootstrap 95% CIs for all 10 specific pathways did not include 0, and *P* < 0.05 (all significant). Comparing the effect sizes of the 10 different mediating pathways (Table 7), the “stress → intimacy → depression” pathway in Model 2 had the highest contribution rate (78.4% in the father group and 70.5% in the pregnant women group), while the “stress → intimacy → disclosure → depression” pathway had a contribution rate of only 9.5%−15.2%. Specifically:

1. In the fathers' group, the “stress → intimacy → depression” pathway had the highest contribution rate (78.4%), representing the primary mediating pathway through which fathers' stress influenced their own depression, whereas the “stress → disclosure → depression” pathway contributed only 9.5%, suggesting that impaired intimacy—rather than reduced disclosure—is the core mechanism through which fathers' stress affects depression.
2. In the pregnant women group, the “stress → intimacy → depression” pathway also had the highest contribution rate (70.5%), representing the primary mediating pathway through which stress affects their own depression, while the “stress → self-disclosure → depression” pathway had a contribution rate of 15.2%.
3. The contribution rates of the chained mediation pathways (P3, P6) were relatively low (12.2 and 14.3%), indicating that the complex transmission effect whereby intimacy influences depression via self-disclosure is relatively weak.
4. In the cross-group analysis, the contribution rates of simple intimacy mediation (P7, P9) (52.4%, 33.3%) were significantly higher than those of chained mediation (P8, P10; 9.5%, 4.8%), suggesting that direct damage to intimate relationships is the absolute core mechanism of the stress spillover effect.

**Table 7:** Standardized indirect effects and contribution rates of specific indirect paths for Model 2 (stress → family cohesion → self-disclosure → depression serial mediation model).

Group	Code	Specific path	Standardized indirect effect	Path contribution rate (%)
Father group	P1	Perceived stress → Fathers' self-disclosure → Depression	0.007	9.5
	P2	Perceived stress → Family cohesion → Depression	0.058	78.4
	P3	Perceived stress → Family cohesion → Fathers' self-disclosure → Depression (serial)	0.009	12.2
Total indirect effect (sum)			0.074	100
Pregnant women group	P4	Perceived stress → Pregnant women's self-disclosure → Depression	0.017	15.2
	P5	Perceived stress → Family cohesion → Depression	0.079	70.5
	P6	Perceived stress → Family cohesion → Pregnant women's self-disclosure → Depression (serial)	0.016	14.3
Total indirect effect (sum)			0.112	100
Cross-group	P7	Pregnant women's perceived stress → Fathers' family cohesion → Fathers' depression	0.011	52.4
	P8	Pregnant women's perceived stress → Fathers' family cohesion → Fathers' self-disclosure → Fathers' depression (serial)	0.002	9.5
	P9	Fathers' perceived stress → Pregnant women's family cohesion → Pregnant women's depression	0.007	33.3
	P10	Fathers' perceived stress → Pregnant women's family cohesion → Pregnant women's self-disclosure → Pregnant women's depression (serial)	0.001	4.8
Total indirect effect (sum)			0.021	100

All indirect effects are standardized. Path contribution rate = (specific indirect effect / total indirect effect) × 100%. “Cohesion” refers to family cohesion; “self-disclosure” refers to the individual's own self-disclosure.

##### 4.5.2.3 Comparison of Model 1 and Model 2

Comparison of the Two Models: Model 1 (Rumination Model) had a lower AIC (60.544 vs. 65.073) and a simpler set of parameters; both the total indirect effect and the proportion of mediation were significantly higher than in Model 2 (Self-Disclosure Model), indicating that rumination is the core mediating mechanism through which stress influences depression, with an effect size approximately 2–3 times that of self-disclosure. However, family closeness was a significant prior mediating variable in both models, suggesting that closeness serves as a key hub through which stress is transmitted to different coping strategies and depressive symptoms. The comparison of fit indices and mediation effects between the two models is shown in Supplementary Table S7.

## 5 Discussion

Using paired data from the third trimester of pregnancy, this study investigated factors associated with prenatal depression among couples in Nanjing. Overall, prenatal depression is a significant health issue faced by both expectant mothers and fathers. Our findings clarify the complex interactions between psychosocial factors in both partners during late pregnancy and depression. Family closeness plays a key role in how both partners cope with stress; it not only directly influences their levels of depression but also affects their depressive mood by influencing their negative thinking and levels of self-disclosure.

### 5.1 Both partners are at high risk for depression in late pregnancy

This study shows that the detection rate of depression among pregnant women was 18.3%, which is relatively consistent with the prevalence rate of perinatal depression in China (19.7%) reported in a meta-analysis (29), Furthermore, the prevalence rate of 16.7% among expectant fathers is higher than the estimate derived from an international meta-analysis (9.8%) (12), but is generally consistent with the levels of depression reported by Tang et al. (20) and Liu et al. (18) among couples in late pregnancy in China. The discrepancies among these studies may stem from differences in screening tools and methods, as well as variations in population characteristics. This suggests that perinatal depression occurs not only in pregnant women but also in fathers, who are similarly at high risk. It is worth noting that although pregnant women's depression scores were slightly higher than those of fathers, fathers' scores for perceived stress and rumination were significantly higher than those of pregnant women, while their scores for self-disclosure, family intimacy, and self-efficacy were all lower than those of pregnant women. This suggests that men may experience greater economic pressure and stress related to role transitions during pregnancy (30, 31), yet they are less likely to actively express their emotions (32), making them more prone to developing negative cognitive processing patterns, which in turn increases their risk of depression. This is consistent with the findings of previous studies, which indicate that fathers with perinatal depression often conceal, normalize, or avoid their distress. This may be because men perceive vulnerability as undermining their masculine identity, and fathers often conceal their symptoms out of a desire to protect their partners and avoid burdening their pregnant wives. These findings support the stress vulnerability model and demonstrate that fathers' mental health should also be a key focus of perinatal screening.

### 5.2 The subject effect is the primary mechanism of couple depression in late pregnancy

This study found that, in both univariate and integrated models, the subject effect was significant, while the object effect was relatively weak; only perceived stress and rumination exhibited limited cross-subject effects in the univariate models. This suggests that perinatal depression in couples is influenced more by individuals' own psychological resources and cognitive styles than by direct effects from their partners, consistent with the findings of Liu et al. based on the APIM framework (18, 33). Based on an APIM analysis of 282 Chinese couples in late pregnancy, Liu et al. found that the effect of prenatal stress on depression follows an “actor-only pattern,” meaning that stress significantly predicts depression in the individual but has no significant effect on the partner. This difference suggest that the partner association may not be direct but may instead operate indirectly through mediating variables such as relationship quality. Chinese couples during pregnancy may be more inclined toward a stress coping style of “bearing negative emotions individually” rather than “coping jointly,” which may weaken the direct cross-actor transmission effect of stress. Among these, perceived stress and rumination are core predictive factors common to both partners, indicating that stress appraisal and negative cognitive processing are important mechanisms underlying the development of perinatal depression, which is consistent with previous research findings (24). Family intimacy was identified as a common protective factor associated with depressive symptoms in both partners. Meanwhile, self-efficacy showed stronger protective associations for pregnant women, while self-disclosure primarily protects fathers, suggesting that different genders may employ distinct coping strategies during psychological adaptation. Future interventions should incorporate gender-specific characteristics to implement targeted management.

Family intimacy was identified as a key pathway linking stress to negative and positive coping patterns, and also as a potential mechanism for cross-subject associations.

This study identified a chain-mediated pathway consistent with the theoretical model: “perceived stress → family closeness → rumination → depression.” Stress was directly associated with depression levels, and was also associated with rumination through reduced family closeness; these pathways were in turn associated with depressive symptoms. This finding is highly consistent with stress coping theory; existing research is generally consistent with the view that perceived stress is associated with depression, both directly and indirectly through resource and coping mechanisms. In the mediation model, family closeness functions more as a relational protective resource, while rumination serves as a proximal cognitive risk factor; this aligns with the notion that “distant stress influences depression through proximal cognitive mechanisms” (34, 35). Among the mediating pathways of negative coping, rumination made the largest contribution, highlighting that impaired relational resources are associated with negative cognitive processing, which in turn is associated with higher depression levels. Furthermore, this study supports the mediating pathway of family closeness for positive coping (self-disclosure): “perceived stress → family closeness → self-disclosure → depression.” In the mediating pathway for positive coping, family intimacy contributed the most, highlighting that close relationships may provide a safe environment for emotional expression. Thus, in the stress coping process examined in this study, rumination explained a larger proportion of the variance in the negative coping pathway, while family intimacy explained a larger proportion in the positive coping pathway. This suggests that reducing rumination and enhancing relational resources may be two complementary intervention targets.

It is worth noting that, compared with the self-disclosure model, the rumination model exhibits greater indirect effects, a higher proportion of mediation, and better model fit indices, indicating that rumination plays a stronger explanatory role in the process by which stress influences depression. This finding is highly consistent with Nolen-Hoeksema's theory of response styles—negative rumination is a relatively stable cognitive style that is more strongly associated with the onset and maintenance of depression than the mere presence or absence of emotional expression. Previous studies have also identified similar patterns among cancer patients and their spouses: the mediating effect of rumination on the relationship between stress and depression is far greater than that of other cognitive variables (33). From the perspective of clinical intervention, this finding has important implications for selecting intervention targets: although enhancing spousal communication and self-disclosure may be associated with improved family relationships, when individual ruminative habits are not simultaneously addressed, negative cognitive processing may persist and depression may be more likely to occur if individual ruminative habits are not simultaneously addressed. In other words, relationship-level interventions (enhancing intimacy, promoting self-disclosure) and individual cognitive-level interventions (reducing rumination) should be pursued in tandem rather than as substitutes for one another. This suggests that, rather than simply increasing emotional expression, priority should be given to helping perinatal couples reduce negative ruminative thinking and lower cognitive vulnerability, which may help mitigate the association between stress and depression. It may also imply that couple-centered psychological interventions should not only focus on emotional communication between partners but that their effectiveness may depend on whether they simultaneously address individual cognitive patterns.

Furthermore, cross-mediation analysis revealed that stress experienced by one partner was indirectly associated with the other partner's depression levels, with this association operating through the partner's perceived family intimacy. This emphasizes that family intimacy may represent a relational pathway through which stress and emotional experiences are linked across partners, rather than merely an individual variable; family intimacy is a crucial mediator of emotional transmission between spouses. This is consistent with previous findings that maternal stress can be indirectly linked to paternal prenatal depression via the father's marital satisfaction, suggesting that relationship quality may indeed mediate cross-individual stress effects (14). Existing research on dyadic coping and stress transmission generally supports this explanation: external stress has been linked to changes in intimate relationships, partner interactions, and satisfaction, and these changes have been associated with the mental health of both partners (36).

### 5.3 Clinical significance

This study suggests that perinatal psychological interventions should move beyond the traditional model of focusing solely on pregnant women and instead treat couples as joint targets of intervention. On the one hand, screening for stress perception and rumination should be strengthened to identify high-risk families as early as possible; on the other hand, the spillover effects of stress within the family should be reduced by enhancing communication between spouses and improving family intimacy. Furthermore, implementing gender-specific interventions—such as enhancing self-efficacy in pregnant women and strengthening stress management and emotional expression training for fathers—can help further improve the effectiveness of perinatal mental health interventions.

## 6 Limitations

This study still has certain limitations. First, this study employed a cross-sectional design, with all variables collected at a single point in time, making it impossible to infer causal relationships among the variables. Although this study constructed a chained mediation model of “stress → family closeness → coping style → depression” based on stress coping theory and statistically tested the significance of each path, cross-sectional data can, by their very nature, only reflect associations between variables and cannot confirm temporal sequence. For example, individuals with higher levels of depression may be more inclined to interpret their stress negatively, underestimate family closeness, and exhibit stronger rumination due to low mood—meaning that reverse causality is also a possibility. Therefore, the mediating pathways revealed in this study still need to be validated through a longitudinal tracking design. Furthermore, as the study included only samples from the third trimester, it remains unclear whether the findings are applicable to the first trimester, second trimester, or postpartum period. Additionally, since the participants were recruited from a single-center hospital, the representativeness of the sample remains limited. Future research could adopt multi-center, multi-regional sampling strategies and combine them with longitudinal designs to track dynamic changes in the same cohort across different perinatal stages, thereby testing the stability of the chained mediation pathways identified in this study across time periods and their generalizability across populations. Second, all variables in this study were measured using self-report questionnaires, which may introduce various forms of bias. Future studies may consider incorporating multi-method, multi-source measurement strategies—such as clinical diagnostic interviews, observational methods, or spousal reports—to reduce the impact of self-report bias on research conclusions. This study used the PHQ-9 for depression screening rather than a clinical diagnostic tool; therefore, the results still require further validation through clinical assessment. Although this study included variables such as parity, maternal age, pregnancy complications, and history of adverse pregnancy and childbirth outcomes as covariates in the multiple linear regression model to control for their confounding effects, due to the cross-sectional design and sample size limitations, the study was unable to perform rigorous matching or stratified sampling for these factors during the recruitment phase. Future studies may consider using propensity score matching, stratified sampling, or a prospective cohort design to control for parity, age, and pregnancy risk level at the recruitment stage, thereby further validating the robustness of the findings of this study. Furthermore, this study did not include variables such as social support, marital satisfaction, and biological indicators. These factors may be associated with the variables of interest in this study—perceived stress, rumination, self-disclosure, and depression—and their exclusion may have led to bias in the estimation of model parameters. Future research should incorporate a more comprehensive set of covariates. A longitudinal design could be employed in future studies to further refine the theoretical model of perinatal depression in couples.

## 7 Conclusion

Depression among couples in late pregnancy is primarily characterized by subject-based associations, with rumination and perceived stress serving as core predictors of depression, while family intimacy and adaptability were identified as important protective correlates. The partner associations of psychosocial factors between spouses were primarily indirect—operating through the association between one spouse's stress and the partner's perceived family intimacy. The complete chain of pathways “stress → reduced family intimacy → increased rumination and reduced self-disclosure → worsening depression” holds true for both partners, with rumination serving as a stronger cognitive mediating mechanism than self-disclosure. Clinical interventions should focus primarily on individual psychological adjustment to reduce rumination in both the expectant mother and the father, while simultaneously emphasizing the improvement of couple intimacy, thereby jointly promoting the alleviation of negative emotions in both partners.

## Data Availability

The original contributions presented in the study are included in the article/Supplementary material, further inquiries can be directed to the corresponding author.

## Supplementary material

Supplementary Table S1 Paired-samples *t*-test results comparing scale scores between partners.

Supplementary Table S2 Full linear regression results for depression scores among pregnant women (hierarchical models).

Supplementary Table S3 Full linear regression results for depression scores among fathers (hierarchical models).

Supplementary Table S4 Univariate APIM results: actor and partner effects for all psychosocial variables.

Supplementary Table S5 Standardized path coefficients for Model 1 (stress → family cohesion → rumination → depression).

Supplementary Table S6 Standardized path coefficients for Model 2 (stress → family cohesion → self-disclosure → depression).

Supplementary Table S7 Comparison of model fit indices and mediation effects between Model 1 (mediator: rumination) and Model 2 (mediator: self-disclosure).

## Appendix

**APPENDIX A:** Univariate analysis results.

Mothers (*n* = 1,331)			Fathers (*n* = 1,331)		
Variable	*F*	*P*	Variable	*F*	*P*
Maternal age	2.711	0.044	Paternal occupation	3.967	0.019
Maternal education	7.428	< 0.001	Paternal alcohol consumption	5.561	0.004
Family monthly income	3.194	0.041	Paternal sleep satisfaction	101.927	< 0.001
Maternal marital quality	20.273	< 0.001	Paternal marital quality	37.584	< 0.001
Maternal preparedness for newborn care	41.722	< 0.001	Paternal preparedness for newborn care	50.451	< 0.001
Maternal sleep satisfaction	170.653	< 0.001	Residence	3.618	0.027
Maternal concern about body image change	11.569	< 0.001	Maternal marital quality	2.992	0.03
Paternal sleep satisfaction	3.906	0.02	Maternal preparedness for newborn care	9.487	< 0.001
Paternal marital quality	5.811	0.003	Maternal sleep satisfaction	4.894	0.008
Planned pregnancy (mother)	12.62	< 0.001	Planned pregnancy (father)	9.623	0.002
Planned delivery mode	6.755	0.009			
Maternal chronic disease	5.048	0.025			
Planned pregnancy (father)	10.561	0.001			
