# Supplementary material for: Analysis of the subject-object effects and chain mediation of psychosocial factors in couples during late pregnancy on depression: a cross-sectional study of pregnant women and their spouses
Source: Front Public Health. 2026 Sep 28;14:1948517. doi: 10.3389/fpubh.2026.1948517 (PMC13655107; doi:10.3389/fpubh.2026.1948517)
Supplement: Supplementary file 1 [file Supplementary_file_1.docx]

| **Table S1. Paired samples test** | | | | | | |
| --- | --- | --- | --- | --- | --- | --- |
| Pair | Mean Difference | Std. Deviation | Std. Error Mean | t | Sig. |  |
| Pair 1 | Pregnant women's PHQ‑9 – Fathers' PHQ‑9 | 0.222 | 3.56 | 0.098 | 2.272 | 0.023 |
| Pair 2 | Pregnant women's perceived stress – Fathers' perceived stress | –1.305 | 9.542 | 0.262 | –4.989 | <0.001 |
| Pair 3 | Pregnant women's self‑disclosure – Fathers' self‑disclosure | 8.968 | 12.263 | 0.336 | 26.678 | <0.001 |
| Pair 4 | Pregnant women's self‑efficacy – Fathers' self‑efficacy | 0.817 | 7.585 | 0.208 | 3.928 | <0.001 |
| Pair 5 | Pregnant women's family cohesion – Fathers' family cohesion | 1.677 | 11.423 | 0.313 | 5.356 | <0.001 |
| Pair 6 | Pregnant women's family adaptability – Fathers' family adaptability | 1.286 | 10.072 | 0.276 | 4.659 | <0.001 |
| Pair 7 | Pregnant women's rumination – Fathers' rumination | –4.183 | 12.876 | 0.353 | –11.851 | <0.001 |

| **Table S2. Linear regression results for depression scores among pregnant women** | | | | | | | |
| --- | --- | --- | --- | --- | --- | --- | --- |
| Variable | Unstandardized Coefficients |  | Standardized Coefficients | t | p | Collinearity Diagnostics | |
|  | Beta | Std. Error | Beta |  |  | VIF | Tolerance |
| Model1 |  |  |  |  |  |  |  |
| (Constant) | 3.848 | 0.449 |  | 8.562 | <0.001 |  |  |
| High school / technical secondary school | –1.863 | 0.552 | –0.127 | –3.374 | 0.001 | 2.465 | 0.406 |
| College / bachelor's degree | –2.145 | 0.437 | –0.348 | –4.910 | <0.001 | 8.718 | 0.115 |
| Graduate degree or above | –2.172 | 0.454 | –0.324 | –4.789 | <0.001 | 7.908 | 0.126 |
| Sleep quality: fair | 1.679 | 0.135 | 0.308 | 12.4 | <0.001 | 1.065 | 0.939 |
| Sleep quality: dissatisfied | 5.23 | 0.317 | 0.407 | 16.477 | <0.001 | 1.057 | 0.946 |
| Concern about appearance: moderate | –0.547 | 0.145 | –0.100 | –3.783 | <0.001 | 1.205 | 0.83 |
| Concern about appearance: not concerned | –1.130 | 0.222 | –0.134 | –5.086 | <0.001 | 1.195 | 0.837 |
| R² = 0.236; Adjusted R² = 0.232; F change = 58.26; Sig. F change <0.001 | | | | | | | |
| Model2 |  |  |  |  |  |  |  |
| (Constant) | 0.474 | 0.825 |  | 0.574 | 0.566 |  |  |
| High school / technical secondary school | –1.027 | 0.46 | –0.070 | –2.232 | 0.026 | 2.489 | 0.402 |
| College / bachelor's degree | –0.983 | 0.369 | –0.160 | –2.665 | 0.008 | 9.033 | 0.111 |
| Graduate degree or above | –0.911 | 0.385 | –0.136 | –2.364 | 0.018 | 8.291 | 0.121 |
| Sleep quality: fair | 0.892 | 0.117 | 0.163 | 7.594 | <0.001 | 1.165 | 0.858 |
| Sleep quality: dissatisfied | 3.47 | 0.274 | 0.27 | 12.674 | <0.001 | 1.143 | 0.875 |
| Concern about appearance: moderate | –0.278 | 0.121 | –0.051 | –2.293 | 0.022 | 1.227 | 0.815 |
| Concern about appearance: not concerned | –0.422 | 0.187 | –0.050 | –2.256 | 0.024 | 1.231 | 0.812 |
| Fathers' rumination total | 0.011 | 0.005 | 0.044 | 2.159 | 0.031 | 1.024 | 0.976 |
| Pregnant women's perceived stress | 0.054 | 0.01 | 0.153 | 5.487 | <0.001 | 1.965 | 0.509 |
| Pregnant women's self‑efficacy | –0.034 | 0.013 | –0.073 | –2.702 | 0.007 | 1.817 | 0.55 |
| Pregnant women's rumination total | 0.103 | 0.007 | 0.337 | 13.932 | <0.001 | 1.47 | 0.68 |
| Family cohesion and adaptability | –0.027 | 0.009 | –0.080 | –2.979 | 0.003 | 1.822 | 0.549 |
| R² = 0.476; Adjusted R² = 0.471; F change = 121.053; Sig. F change <0.001 | | | | | | | |

*Table S2 Notes:*

*Dependent variable: maternal PHQ-9 score.*

*Reference groups for categorical variables were: education = middle school or below; sleep satisfaction = satisfied; concern about body image change = concerned.*

*Model 1 adjusted for demographic variables only. Model 2 additionally adjusted for paternal rumination total score, maternal perceived stress total score, maternal self-efficacy total score, maternal rumination total score, and family cohesion/adaptability total score (FACES II).*

*VIF = Variance Inflation Factor; all VIFs were < 10, indicating no severe multicollinearity.*

| **Table S3. Linear regression results for depression scores among fathers** | | | | | | | |
| --- | --- | --- | --- | --- | --- | --- | --- |
| Variable | Unstandardized Coefficients |  | Standardized Coefficients | t | p | Collinearity Diagnostics | |
|  | Beta | Std. Error | Beta |  |  | VIF | Tolerance |
| Model1 |  |  |  |  |  |  |  |
| (Constant) | 1.206 | 0.1 |  | 12.01 | <0.001 |  |  |
| Father's occupation: part‑time | 1.322 | 0.589 | 0.055 | 2.243 | 0.025 | 1.006 | 0.994 |
| Father's sleep quality: fair | 1.312 | 0.142 | 0.236 | 9.27 | <0.001 | 1.079 | 0.927 |
| Father's sleep quality: dissatisfied | 4.33 | 0.443 | 0.243 | 9.773 | <0.001 | 1.026 | 0.975 |
| Father's marital quality: good | 0.898 | 0.205 | 0.111 | 4.374 | <0.001 | 1.074 | 0.931 |
| Father's readiness for newborn care: fair | 1.094 | 0.182 | 0.154 | 6.017 | <0.001 | 1.09 | 0.917 |
| Father's readiness for newborn care: inadequate | 4.925 | 0.99 | 0.123 | 4.975 | <0.001 | 1.011 | 0.989 |
| Residence: rented house in city | –0.457 | 0.186 | –0.061 | –2.463 | 0.014 | 1.013 | 0.988 |
| Pregnant women's readiness: fair | 0.438 | 0.148 | 0.074 | 2.963 | 0.003 | 1.025 | 0.976 |
| Pregnant women's readiness: inadequate | 1.946 | 0.732 | 0.066 | 2.658 | 0.008 | 1.009 | 0.991 |
| R² = 0.205; Adjusted R² = 0.199; F change = 37.82; Sig. F change <0.001 | | | | | | | |
| Model2 |  |  |  |  |  |  |  |
| (Constant) | –0.608 | 0.646 |  | –0.941 | 0.347 |  |  |
| Father's occupation: part‑time | 1.005 | 0.526 | 0.042 | 1.911 | 0.056 | 1.009 | 0.992 |
| Father's sleep quality: fair | 0.833 | 0.129 | 0.15 | 6.47 | <0.001 | 1.126 | 0.888 |
| Father's sleep quality: dissatisfied | 3.014 | 0.401 | 0.169 | 7.511 | <0.001 | 1.06 | 0.943 |
| Father's marital quality: good | 0.481 | 0.186 | 0.06 | 2.587 | 0.01 | 1.108 | 0.902 |
| Father's readiness for newborn care: fair | 0.46 | 0.166 | 0.065 | 2.777 | 0.006 | 1.141 | 0.877 |
| Father's readiness for newborn care: inadequate | 4.373 | 0.883 | 0.109 | 4.951 | <0.001 | 1.013 | 0.987 |
| Residence: rented house in city | –0.497 | 0.166 | –0.066 | –3.003 | 0.003 | 1.013 | 0.987 |
| Pregnant women's readiness: fair | 0.264 | 0.132 | 0.044 | 2.002 | 0.045 | 1.032 | 0.969 |
| Pregnant women's readiness: inadequate | 1.709 | 0.653 | 0.058 | 2.618 | 0.009 | 1.011 | 0.989 |
| Fathers' perceived stress total | 0.146 | 0.01 | 0.382 | 15.159 | <0.001 | 1.332 | 0.751 |
| Fathers' self‑disclosure total | –0.014 | 0.007 | –0.051 | –2.155 | 0.031 | 1.155 | 0.866 |
| Fathers' family cohesion and adaptability | –0.029 | 0.009 | –0.086 | –3.349 | 0.001 | 1.381 | 0.724 |
| R² = 0.370; Adjusted R² = 0.365; F change = 115.372; Sig. F change <0.001 | | | | | | | |
| Model3 |  |  |  |  |  |  |  |
| (Constant) | –2.041 | 0.642 |  | –3.181 | 0.002 |  |  |
| Father's occupation: part‑time | 1.027 | 0.508 | 0.043 | 2.021 | 0.043 | 1.009 | 0.991 |
| Father's sleep quality: fair | 0.786 | 0.125 | 0.141 | 6.307 | <0.001 | 1.127 | 0.887 |
| Father's sleep quality: dissatisfied | 2.78 | 0.388 | 0.156 | 7.158 | <0.001 | 1.064 | 0.94 |
| Father's marital quality: good | 0.448 | 0.18 | 0.056 | 2.497 | 0.013 | 1.108 | 0.902 |
| Father's readiness for newborn care: fair | 0.381 | 0.16 | 0.054 | 2.377 | 0.018 | 1.143 | 0.875 |
| Father's readiness for newborn care: inadequate | 3.696 | 0.856 | 0.092 | 4.317 | <0.001 | 1.02 | 0.98 |
| Residence: rented house in city | –0.377 | 0.16 | –0.050 | –2.348 | 0.019 | 1.02 | 0.981 |
| Pregnant women's readiness: fair | 0.314 | 0.128 | 0.053 | 2.46 | 0.014 | 1.034 | 0.967 |
| Pregnant women's readiness: inadequate | 1.636 | 0.631 | 0.055 | 2.593 | 0.01 | 1.011 | 0.989 |
| Fathers' perceived stress total | 0.107 | 0.01 | 0.282 | 10.67 | <0.001 | 1.569 | 0.637 |
| Fathers' self‑disclosure total | –0.010 | 0.006 | –0.036 | –1.568 | 0.117 | 1.161 | 0.862 |
| Fathers' family cohesion and adaptability | –0.023 | 0.008 | –0.068 | –2.737 | 0.006 | 1.388 | 0.72 |
| Fathers' rumination total | 0.062 | 0.006 | 0.24 | 9.726 | <0.001 | 1.37 | 0.73 |
| R² = 0.412; Adjusted R² = 0.407; F change = 94.597; Sig. F change <0.001 | | | | | | | |

*Table S3 Notes:*

*Dependent variable: paternal PHQ-9 score.*

*Reference groups for categorical variables were: occupation = employed; sleep satisfaction = satisfied; marital quality = very good; preparedness for newborn care = fully prepared; residence = own house in urban area; maternal preparedness for newborn care = fully prepared (when included as a covariate in paternal models).*

*Hierarchical linear regression was performed to examine the independent effects of psychosocial factors on paternal depressive symptoms after adjusting for covariates.*

*Covariates (entered in Model 1): paternal occupation, paternal sleep satisfaction, paternal marital quality, paternal preparedness, maternal preparedness, and residence.*

*Key independent variables (entered in Model 2 & 3): paternal perceived stress, paternal self-disclosure, family cohesion/adaptability, and paternal rumination.*

*ΔR² from Model 1 to Model 2 = 0.165; ΔR² from Model 2 to Model 3 = 0.042 (both p < 0.001). The attenuation of the effect of paternal self-disclosure after entering rumination (Model 3) suggests a mediating or overlapping mechanism between these two cognitive/behavioral factors.*

*VIF = Variance Inflation Factor; all VIFs were < 10, indicating no severe multicollinearity.*

| **Table S4. Univariate model results (actor and partner effects)** | | | | | |
| --- | --- | --- | --- | --- | --- |
| Model | Independent variable | Actor effect β | p | Partner effect β | p |
| Model 1 | Pregnant women's perceived stress | 0.503 | <0.001 | 0.04 | 0.095 |
|  | Fathers' perceived stress | 0.515 | <0.001 | 0.052 | 0.028 |
| Model 2 | Pregnant women's self‑disclosure | –0.321 | <0.001 | –0.050 | 0.061 |
|  | Fathers' self‑disclosure | –0.219 | <0.001 | –0.026 | 0.321 |
| Model 3 | Pregnant women's self‑efficacy | –0.383 | <0.001 | –0.030 | 0.261 |
|  | Fathers' self‑efficacy | –0.267 | <0.001 | –0.019 | 0.45 |
| Model 4 | Pregnant women's family cohesion | –0.398 | <0.001 | –0.027 | 0.313 |
|  | Fathers' family cohesion | –0.353 | <0.001 | –0.028 | 0.283 |
| Model 5 | Pregnant women's family adaptability | –0.427 | <0.001 | –0.043 | 0.105 |
|  | Fathers' family adaptability | –0.338 | <0.001 | –0.016 | 0.514 |
| Model 6 | Pregnant women's rumination | 0.555 | <0.001 | 0.037 | 0.126 |
|  | Fathers' rumination | 0.472 | <0.001 | 0.067 | 0.003 |
| Model 7 | Pregnant women's family cohesion and adaptability | –0.426 | <0.001 | –0.032 | 0.225 |
|  | Fathers' family cohesion and adaptability | –0.359 | <0.001 | –0.020 | 0.439 |

**Table S5. Standardised path coefficients for the serial mediation model**

| Path | Father Group β | Pregnant Women Group β |
| --- | --- | --- |
| Within‑group paths |  |  |
| Perceived stress → Family cohesion | –0.428*** | –0.538*** |
| Family cohesion → Rumination | –0.144*** | –0.214*** |
| Perceived stress → Rumination | 0.429*** | 0.397*** |
| Rumination → Depression | 0.271*** | 0.389*** |
| Family cohesion → Depression | –0.119*** | –0.092*** |
| Perceived stress → Depression | 0.332*** | 0.252*** |
| Cross‑subject paths |  |  |
| Pregnant women's perceived stress → Fathers' family cohesion | –0.081** | — |
| Fathers' perceived stress → Pregnant women's family cohesion | — | –0.048* |
| Pregnant women's perceived stress → Fathers' depression | 0.024 (p = 0.296) | — |
| Fathers' perceived stress → Pregnant women's depression | — | 0.053* |

*Note: ***p < 0.001, **p < 0.01, *p < 0.05.*

**Table S6. Path coefficients for the serial mediation model of stress → family cohesion → self‑disclosure → depression**

| Path | Father Group β | Pregnant Women Group β |
| --- | --- | --- |
| Perceived stress → Family cohesion | –0.428*** | –0.538*** |
| Family cohesion → Self‑disclosure | 0.293*** | 0.392*** |
| Perceived stress → Self‑disclosure | –0.103*** | –0.224*** |
| Self‑disclosure → Depression | –0.068** | –0.075** |
| Family cohesion → Depression | –0.136*** | –0.147*** |
| Perceived stress → Depression | 0.440*** | 0.391*** |
| Cross‑subject paths |  |  |
| Pregnant women's perceived stress → Fathers' family cohesion | –0.081** | — |
| Fathers' perceived stress → Pregnant women's family cohesion | — | –0.048* |
| Pregnant women's family cohesion → Fathers' self‑disclosure | 0.049 (p = 0.062) | — |
| Fathers' family cohesion → Pregnant women's self‑disclosure | — | –0.030 (p = 0.202) |
| Pregnant women's perceived stress → Fathers' depression (direct) | 0.028 (p = 0.226) | — |
| Fathers' perceived stress → Pregnant women's depression (direct) | — | 0.045 (p = 0.055) |

*Note:* ***p < 0.001, **p < 0.01, *p < 0.05.

**Table S7. Comparison of model fit indices and mediation effects between Model 1 (mediator: rumination) and Model 2 (mediator: self‑disclosure)**

| Comparison Dimension | Model 1 (Mediator: Rumination) | Model 2 (Mediator: Self‑disclosure) |
| --- | --- | --- |
| Father group |  |  |
| Total indirect effect | 0.184 | 0.074 |
| Mediation proportion | 35.70% | 14.40% |
| Pregnant women group |  |  |
| Total indirect effect | 0.249 | 0.112 |
| Mediation proportion | 49.70% | 22.30% |
| Dominant pathway in father group | Perceived stress → Rumination → Depression (63.8%) | Perceived stress → Cohesion → Depression (78.4%) |
| Dominant pathway in pregnant women group | Perceived stress → Rumination → Depression (62.1%) | Perceived stress → Cohesion → Depression (70.5%) |
| Mediation type in cross‑group paths | Complete / Partial | Complete / Complete |
| Fit indices |  |  |
| CMIN/DF | 0.568 | 0.845 |
| RMSEA | 0 | 0 |
| RMSEA 90% CI | (0.000, 0.020) | (0.000, 0.032) |
| CFI | 1 | 1 |
| TLI | 1.004 | 1.002 |
| GFI | 0.999 | 0.999 |
| AIC | 60.544 | 65.073 |
| Number of estimated parameters (NPAR) | 28 | 30 |

*Note: “Cohesion” refers to family cohesion. Model 1 includes rumination as the mediator; Model 2 includes self‑disclosure as the mediator. Complete mediation indicates that the direct effect is non‑significant (p > 0.05) while the indirect effect is significant; partial mediation indicates both direct and indirect effects are significant.*
